# Enhancing Deer Sous Vide Meat Shelf Life and Safety with *Eugenia caryophyllus* Essential Oil against *Salmonella enterica*

**DOI:** 10.3390/foods13162512

**Published:** 2024-08-12

**Authors:** Miroslava Kačániová, Stefania Garzoli, Anis Ben Hsouna, Zhaojun Ban, Joel Horacio Elizondo-Luevano, Maciej Ireneusz Kluz, Rania Ben Saad, Peter Haščík, Natália Čmiková, Božena Waskiewicz-Robak, Ján Kollár, Alessandro Bianchi

**Affiliations:** 1 Institute of Horticulture, Faculty of Horticulture and Landscape Engineering, Slovak University of Agriculture, Trieda Andreja Hlinku 2, 94976 Nitra, Slovakia; n.cmikova@gmail.com; 2 School of Medical & Health Sciences, University of Economics and Human Sciences in Warsaw, Okopowa 59, 01043 Warszawa, Poland; m.kluz@vizja.pl (M.I.K.); b.waszkiewicz-robak@vizja.pl (B.W.-R.); 3 Department of Chemistry and Technologies of Drug, Sapienza University, P. le Aldo Moro, 5, 00185 Rome, Italy; stefania.garzoli@uniroma1.it; 4 Laboratory of Biotechnology and Plant Improvement, Centre of Biotechnology of Sfax, B.P “1177”, Sfax 3018, Tunisia; benhsounanis@gmail.com (A.B.H.); raniabensaad@gmail.com (R.B.S.); 5 Department of Environmental Sciences and Nutrition, Higher Institute of Applied Sciences and Technology of Mahdia, University of Monastir, Monastir 5000, Tunisia; 6 School of Biological and Chemical Engineering, Zhejiang University of Science and Technology, Zhejiang Provincial Key Laboratory of Chemical and Biological Processing Technology of Farm Products, Zhejiang Provincial Collaborative Innovation Center of Agricultural Biological Resources Biochemical Manufacturing, Hangzhou 310023, China; banzhaojun@zust.edu.cn; 7 Faculty of Agronomy, Universidad Autónoma de Nuevo León (UANL), Av. Francisco Villa S/N, Col. Ex Hacienda el Canadá, General Escobedo, Nuevo León 66050, Mexico; joel.elizondolv@uanl.edu.mx; 8 Institute of Food Technology, Faculty of Biotechnology and Food Sciences, Slovak University of Agriculture, Trieda Andreja Hlinku 2, 94976 Nitra, Slovakia; peter.hascik@uniag.sk; 9 Institute of Landscape Architecture, Faculty of Horticulture and Landscape Engineering, Slovak University of Agriculture, Tulipánová 7, 94976 Nitra, Slovakia; jan.kollar@uniag.sk; 10 Department of Agriculture, Food and Environment, University of Pisa, Via del Borghetto 80, 56124 Pisa, Italy; alessandro.bianchi@phd.unipi.it

**Keywords:** clove essential oil, chemical composition, antimicrobial activity, antibiofilm activity, insecticidal activity, game meat, pathogenic bacteria, chili cook

## Abstract

Modern lifestyles have increased the focus on food stability and human health due to evolving industrial goals and scientific advancements. Pathogenic microorganisms significantly challenge food quality, with *Salmonella enterica* and other planktonic cells capable of forming biofilms that make them more resistant to broad-spectrum antibiotics. This research examined the chemical composition and antibacterial and antibiofilm properties of the essential oil from *Eugenia caryophyllus* (ECEO) derived from dried fruits. GC-MS analyses identified eugenol as the dominant component at 82.7%. Additionally, the study aimed to extend the shelf life of sous vide deer meat by applying a plant essential oil and inoculating it with *S. enterica* for seven days at 4 °C. The essential oil demonstrated strong antibacterial activity against *S. enterica.* The ECEO showed significant antibiofilm activity, as indicated by the MBIC crystal violet test results. Data from MALDI-TOF MS analysis revealed that the ECEO altered the protein profiles of bacteria on glass and stainless-steel surfaces. Furthermore, the ECEO was found to have a beneficial antibacterial effect on *S. enterica*. In vacuum-packed sous vide red deer meat samples, the anti-*Salmonella* activity of the ECEO was slightly higher than that of the control samples. These findings underscore the potential of the ECEO’s antibacterial and antibiofilm properties in food preservation and extending the shelf life of meat.

## 1. Introduction

The preservation of food product quality during storage is a crucial step for reducing food waste and enhancing sustainability [1,2]. Ensuring food safety is equally important, as it protects consumers from potential health hazards caused by microbial contamination [3]. Enhancing food longevity with modern preservation techniques, including the application of natural essential oils, helps ensure the retention of nutritional value and safety in food products [3,4,5]. This approach not only minimizes waste but also supports sustainable practices in the food industry, aligning with modern health and environmental goals [6].

During the production and storage of food, certain bacterial contaminants have the ability to proliferate or endure. Salmonellosis, one of the most common food-borne infections worldwide, is caused by the presence of *Salmonella*, posing a significant risk associated with food products [7]. *Salmonella*’s adaptability to various temperatures and its robust heat-stress resistance enable it to evade host defenses and establish infections. Furthermore, thermal stress responses can activate genes associated with virulence and overall stress resilience [8].

Foods, whether raw or processed, are susceptible to contamination during manufacturing, sale, and distribution processes [9]. Consequently, the food industry requires preservatives to inhibit the growth of microorganisms responsible for food spoilage [10]. Before the early 1990s, there was a limited amount of research on the impact of essential oils (EOs) in food, even though EOs were included in a few commonly used food preservatives [11]. Generally, bacteria are more vulnerable to the antibacterial action of EOs when food pH is lowered, storage temperature is raised, and packing oxygen content is increased. The antibacterial activity of EOs may be restricted by the physical properties of food. Moreover, research has shown that a number of EOs are more effective as bactericidal agents than commonly used preservatives in meat applications [12].

Clove oil has long been utilized in the food industry both as a flavoring agent and for its antimicrobial properties. Its biological activities include antibacterial, antifungal, insecticidal, and antioxidant effects [13]. In addition to its use in food, clove oil serves as an antiseptic for treating oral infections [14]. This essential oil effectively inhibits the growth of molds, yeasts, and bacteria [15]. It has demonstrated efficacy against *Listeria monocytogenes* and *Salmonella* Enteritidis in both tryptone soy broth and cheese [16]. Essential oils are known for their strong anti-biofilm properties, especially when used in combination with antibacterial agents [17]. Plants are a rich source of secondary metabolites like tannins, terpenoids, alkaloids, and flavonoids, many of which have shown antibacterial properties in various studies [18].

Synthetic chemical products from various toxicological classes are used to control insects in stored grains. Although these treatments are highly effective, frequent use can lead to several issues, including increased production costs, food residue buildup, insect resistance, harm to human health, and environmental contamination [19]. The plants can have their parts processed into powders, extracts, or oils for application. As noted by Mazzonetto and Vendramim [20], these products are cost-effective, readily accessible, easy to apply without the need for specialized personnel, and are environmentally and medically safe, posing no adverse effects.

This study examined the heat resistance of five different *Salmonella* strains in teriyaki-marinated chicken breasts using a sous vide method. The chicken breasts, packaged and inoculated, were submerged entirely in a circulating water bath. Temperature settings of 55, 57.5, or 60 °C were achieved within an hour and maintained for specific durations. Based on linear regression analysis, *Salmonella* D-values ranged from 47.65 min at 55 °C to 7.48 min at 60 °C in chicken breast samples. Post-marination, the bacteria exhibited increased susceptibility to heat lethality, which is critical for ensuring the microbiological safety of sous vide-processed marinated meats [21]. To enhance the efficacy of sous vide, EOs were combined with *S. enterica* in another experiment. Samples were vacuum-sealed, inoculated with *S. enterica*, and cooked sous vide at temperatures ranging from 50 to 65 °C for specified periods. After a 20 min heat treatment at 65 °C, significant reductions in bacteria and coliforms were observed in the samples. The integration of EOs with sous vides effectively contributed to the meat’s stabilization and safety [22].

To our knowledge, this is the first study on the use of ECEOs in meat preservation, specifically in game meat. Clove is a highly prized spice used as a food preservative and for a variety of therapeutic reasons. The ECEO and its principal active component, eugenol, indicate antibacterial and antifungal action, aromaticity, and safety as promising and valuable antiseptics in the food sector. The ECEO can help improve the flavor of deer meat. In addition, it can improve the palatability of venison by adding sweetness and tenderness to the meat. However, the effects of the ECEO on venison have never been investigated in detail. The objective of this research was to examine the chemical composition, antimicrobial properties, and effectiveness of the *Eugenia caryophyllus* essential oil against the biofilm formation of *Salmonella enterica in vitro*. Additionally, this study investigated the survival of *S. enterica* inoculated onto red deer meat processed using the sous vide cook–chill method and subsequently stored at 4 °C for 7 days, aiming to extend its shelf life.

## 2. Materials and Methods

### 2.1. Essential Oil Characteristics

The essential oil of clove (*Eugenia caryophyllus*) ECEO used in this research was procured from Hanus s.r.o. in Nitra. It was extracted by steam distillation of dried flower buds sourced from Sri Lanka.

### 2.2. Chemical Analysis of ECEO

The analysis was conducted using a Perkin Elmer Clarus 500 (Waltham, MA, USA) gas chromatograph equipped with a mass spectrometer and a flame ionization detector. A Varian Factor Four VF-5 capillary column (Lambda Life s.r.o., Bratislava, Slovakia)was housed in the GC oven, with helium serving as the carrier gas flowing at a rate of 1 mL/min. The temperature program for the GC oven began with an initial isothermal phase at 60 °C for 2 min, followed by a gradual increase to 220 °C at a rate of 6 °C/min, maintaining the final temperature for 20 min. Mass spectra were obtained in electron impact mode at 70 eV, scanning from 35 to 450 *m*/*z*. Identification of volatile compounds involved comparing their mass spectra with entries in the Wiley 2.2 and Nist 02 databases and determining their linear retention indices (LRIs) relative to C_8_–C_25_ *n*-alkanes analyzed under similar conditions described in the literature. The relative amounts of compounds, expressed as percentages, were calculated by normalizing peak areas without relying on internal standards or any corrective measures. Each experimental run was conducted in triplicate to ensure robustness and consistency of the findings [23,24].

### 2.3. Antimicrobial Activity

#### 2.3.1. Bacteria Strain Preparation

This experiment focused on using *Salmonella enterica* to investigate the effects of sous vide cooking on deer meat and its impact on extending shelf life by reducing microbial counts and antimicrobial activity. The *Salmonella enterica* subsp. *enterica* CCM 4420 strain used was obtained from a microbial collection, the Czech Collection of Microorganisms in Brno, Czech Republic. The bacteria were cultured on Mueller Hinton agar (MHA) from Oxoid in Basingstoke, UK, and were incubated for 24 h at 37 °C. Once the bacterial culture reached an optical density adjusted to the 0.5 McFarland standard (equivalent to 1.5 × 10^8^ CFU/mL), 100 µL of the inoculum was added to samples of deer thigh flesh. To ensure even distribution of the pathogen, the deer meat samples were thoroughly mixed for three minutes at room temperature following inoculation with *Salmonella enterica* [3,22].

#### 2.3.2. Disk Diffusion Method

To assess the antimicrobial efficacy of the ECEO, we employed the disk diffusion method. Bacterial cultures were grown in Mueller–Hinton Broth (MHB, Oxoid, Basingstoke, UK) at 37 °C for 24 h. Following incubation, the bacterial density was adjusted to 0.5 McFarland standard (1.5 × 10^8^ CFU/mL) using distilled water. Subsequently, 100 μL of the bacterial suspension was spread evenly on Mueller–Hinton Agar (MHA, Oxoid, Basingstoke, UK). Sterile 6 mm disks saturated with 10 μL of the ECEO were placed on the agar plates. After incubating at 37 °C for 24 h, the zones of inhibition were measured from three different directions around each disk. Ciprofloxacin (30 µg per disc) was included as the control antibiotic. Each experiment to evaluate antimicrobial activity was conducted in triplicate to ensure a thorough and consistent assessment of results [22,25].

#### 2.3.3. Minimal Inhibitory Concentration (MIC)

Bacterial cultures were incubated for 24 h in Mueller–Hinton broth (MHB, Oxoid, Basingstoke, UK) at 37 °C. The cultures were adjusted to an optical density corresponding to 0.5 McFarland standard and then added in 150 μL volumes to each well of a 96-well microplate. The ECEO was also added in 150 μL volumes to achieve final concentrations ranging from 10 mg/mL to 0.00488 mg/mL. The microplate was then incubated for 24 h at 37 °C. Negative controls consisted of MHB with the ECEO, while positive controls included MHB with bacterial inoculum. Following incubation, absorbance at 570 nm was measured using a Glomax spectrophotometer (Promega Inc., Madison, WI, USA). The MIC_50_ was defined as the lowest EO concentration inhibiting 50% of bacterial growth, and the MIC_90_ as the concentration inhibiting 90% of growth. To ensure accuracy and reliability, the experiment was conducted in triplicate [26].

### 2.4. Research on Biofilm Growth

#### 2.4.1. Crystal Violet Study

Kačániová et al. [25] conducted a comprehensive study on the Minimal Biofilm Inhibitory Concentration (MBIC). Bacterial suspensions were cultured in Mueller–Hinton broth (MHB, Oxoid, Basingstoke, UK) at 37 °C under aerobic conditions throughout the day. After incubation, an inoculum was prepared to achieve an optical density equivalent to the 0.5 McFarland standard. A 96-well microtiter plate was set up by adding 100 μL of the bacteria and 100 μL of the ECEO per well. Starting from the first column, 100 μL of the ECEO was added, followed by a two-fold dilution using a pipette to achieve concentrations ranging from 10 mg/mL to 0.00488 mg/mL. Maximal growth control was maintained using MHB with bacterial inoculum, while MHB with the ECEO served as the negative control. After a 24 h incubation period at 37 °C, the supernatant was discarded, and the wells were washed three times with 250 μL of saline solution before drying at room temperature for 30 min. The wells were then stained with 200 μL of 0.1% *w*/*v* crystal violet for 15 min, followed by several washes with distilled water and subsequent drying. The samples were solubilized with 200 μL of 33% acetic acid, and absorbance at 570 nm was measured using a Glomax spectrophotometer (Promega Inc., Madison, USA). The MBIC was determined as the concentration where the absorbance was equal to or less than the negative control. MBIC_50_ and MBIC_90_ were defined as the concentrations inhibiting 50% and 90% of biofilm development, respectively.

#### 2.4.2. MALDI-TOF MS Biotyper for Biofilm Formation Detection

The Bruker Daltonics MALDI-TOF MicroFlex instrument (Bremen, Germany)was employed to assess protein degradation during biofilm formation. Initially, 100 μL of *S. enterica* bacterial inoculum and 20 mL of MHB were combined in 50 mL polypropylene tubes containing small glass and stainless-steel slides. Experimental tubes were treated with the ECEO to achieve a final concentration of 0.1%, while control tubes remained untreated. Over seven days at 37 °C, tubes were agitated at 170× *g*. Each day, biofilms from glass and steel surfaces were collected using sterile cotton swabs and transferred to target plates. Planktonic cells from untreated control samples were also analyzed. After adding 300 µL of culture material, control bacterial cultures were centrifuged for one minute at 12,000× *g*. Pellets underwent three washes in ultrapure water before being centrifuged again and transferred to target plates for analysis. Reconstituted pellets and swabs (1 μL each) were applied to plates with 10 mg/mL of α-cyano-4-hydroxycinnamic acid matrix. Plates were dried and subjected to MALDI-TOF analysis in linear positive mode, with mass-to-charge ratios calibrated between 2000 and 20,000. Eighteen standard global spectra (MSPs), as described by Kačániová et al. [25,26], were analyzed using automated methods to calculate Euclidean distances and construct dendrograms.

### 2.5. Extending the Shelf Life of Deer Sous Vide Meat

#### 2.5.1. Preparation of Samples of Deer Meat

This study focused on examining deer meat samples obtained from the biceps femoris muscle of a 5-year-old deer originating from Slovak hunting grounds. The analysis of the thigh meat revealed its composition per 100 g: 71.97 g of water, 0.75 g of fat, 21.85 g of protein, and 0.035 g of cholesterol. A total of 4 kg of thigh meat was collected and initially stored in a refrigerator before being transferred to a microbiological laboratory for further analysis. The meat was then sliced into 5 g portions using a sterile knife, resulting in 723 individual samples. These samples were allocated across different time points as follows: three raw deer meat samples on day 0 and 240 samples each on days 1, 7, and 14 for both control and treated groups. Each 5 g portion of deer meat was divided into control and treatment groups. For the treatment group, the meat was mixed with a 1% (*v*/*w*) solution of the ECEO dissolved in sunflower oil. Following this, all samples underwent vacuum packing using a Concept vacuum packer from Chocen, Czech Republic. Control samples were packed in polyethylene bags, while the treatment groups were vacuum-packed after mixing with the ECEO solution.

During the preparation process, 100 µL of *Salmonella enterica* was added to each sample, along with the ECEO solution. Careful precautions were taken to prevent contamination during the brief mixing period, which lasted approximately one minute prior to vacuum sealing. During our trial, we explored various methods for preparing fresh deer meat:

1. Fresh deer meat was stored in polyethylene bags at 4 °C and then cooked at temperatures between 50 °C and 65 °C for 5 to 25 min;
2. Control vacuum: Deer meat, vacuum-sealed in polyethylene bags at 4 °C, underwent cooking in a water bath at temperatures from 50 °C to 65 °C for 5 to 25 min;
3. Essential oil treatment: Deer meat treated with a 1% ECEO solution, vacuum-packed, and kept at 4 °C was cooked in a water bath at temperatures from 50 °C to 65 °C for 5 to 25 min;
4. *Salmonella enterica* contamination: Deer meat inoculated with *Salmonella enterica*, vacuum-packed, stored at 4 °C until exposed, then cooked in a water bath at temperatures from 50 °C to 65 °C for 5 to 25 min;
5. *Salmonella enterica* and essential oil treatment: Deer meat treated with both *Salmonella enterica* and a 1% ECEO solution, vacuum-packed, stored at 4 °C, and subsequently cooked in a water bath at temperatures from 50 °C to 65 °C for 5 to 25 min.

On day zero, raw deer meat samples were processed as controls. These samples were mixed with either the ECEO or *Salmonella enterica* and allowed to rest for 24 h before undergoing sous vide cooking using the CASO SV1000 machine from Arnsberg, Germany. The meat was packed in polyethylene high-barrier bags known for their durability, resistance to moisture, and capability to withstand temperatures ranging from −30 °C to +100 °C. These bags are specifically designed without plasticizers like bisphenol A or microplastics, ensuring food safety during prolonged refrigeration.

#### 2.5.2. Microbial Analyses

Microbiological evaluations were conducted periodically throughout the experiment. Following a 24 h storage period at 4 °C, the samples underwent heat treatment and were then assessed at scheduled intervals. Initially, 5 g of red deer meat samples were placed in sterile stomacher bags and diluted with 45 mL of peptone water to achieve a 1:10 dilution ratio. The samples were homogenized using a stomacher apparatus for 20 min. After homogenization, 0.1 mL aliquots from appropriate dilutions were spread onto standard plate count agar medium and incubated in a shaking incubator for 30 min. For culturing coliform bacteria, Violet Red Bile Lactose Agar (VRBL; Oxoid, Basingstoke, UK) was utilized and incubated at 37 °C for 24 to 48 h. Plate Count Agar (PCA; Oxoid, Basingstoke, UK) was employed for Total Viable Count (TVC) and incubated at 30 °C for 48 to 72 h. Viable counts were determined based on visible growth on these media. *Salmonella* spp. were detected by culturing on Xylose Lysine Deoxycholate Agar (XLD; Oxoid, Basingstoke, UK) and incubating for 24 to 48 h.

#### 2.5.3. Identification of Microorganisms Using Mass Spectrometry

Microorganisms derived from deer thigh tissue samples were identified using the MALDI-TOF MS Biotyper system from Bruker Daltonics in Bremen, Germany, employing established reference libraries. To prepare the matrix solution, an initial stock was created comprising 50% acetonitrile, 47.5% water, and 2.5% trifluoroacetic acid. This stock solution was formulated by combining 500 µL of pure acetonitrile, 475 µL of filtered water, and 25 µL of 10% trifluoroacetic acid. Subsequently, the “HCCA matrix solution” was prepared in a 250 µL Eppendorf flask, thoroughly mixed with the organic solvent, and sourced from Aloqence Science in Vrable, Slovakia, based on prior guidance [22]. Eight distinct colonies from the Petri dishes were then processed accordingly. Biological material from these colonies was transferred into an Eppendorf flask with 300 µL of distilled water, mixed thoroughly, and centrifuged at 10,000× *g* for two minutes using a ROTOFIX 32A centrifuge from ITES in Vranov, Slovakia. Following centrifugation, 900 µL of ethanol was added, and after removal of the supernatant, the pellet was air-dried at room temperature (20 °C). Finally, 30 µL of 70% formic acid and 30 µL of acetonitrile were added to the pellet. Scores obtained from the MALDI-TOF analysis were interpreted based on the following criteria: scores below 1.700 were considered unreliable, scores between 2.300 and 3.000 indicated highly probable species identification, scores between 2.000 and 2.299 suggested genus identification with potential species identification, and scores between 1.700 and 1.999 indicated likely genus identification.

### 2.6. Statistic Analysis

Each evaluation was performed three times, and the findings are expressed as mean values ± the standard deviation (SD). Statistical analysis was conducted using a one-way ANOVA (CoStat version 6.451, CoHort Software, Pacific Grove, CA, USA) followed by Duncan’s multiple range test (MRT), with significance set at *p* ≤ 0.05 for sample differentiation.

Graphical representation was generated using JMP Pro 17.0 software (SAS Institute, Cary, NC, USA).

## 3. Results

### 3.1. Chemical Composition of ECEO

**Figure 1:**
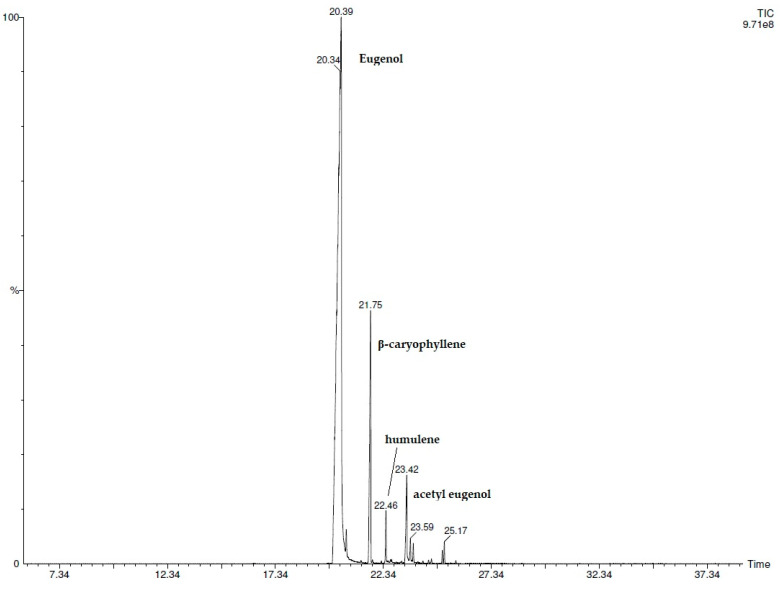
GC-MS chromatogram of the ECEO.

The composition of the ECEO was analyzed using GC-MS methodology. A total of 11 compounds were detected, as detailed in Table 1. Eugenol, identified as a predominant phenolic compound, constituted 82.7% of the composition, with β-caryophyllene (9.9%), acetyl eugenol (3.4%), and humulene (1.4%) identified as the other major constituents. Additionally, a chromatogram is depicted in Figure 1 to illustrate the compound’s elution profiles.

### 3.2. Antimicrobial Activity of ECEO

Table 2 presents the results of the antimicrobial activity evaluation of the ECEO using the disc diffusion method and minimal inhibitory concentration (MIC). The ECEO exhibited significant antimicrobial activity against *S. enterica*, as evidenced by a zone of inhibition measuring 15.67 mm. In contrast, the antibiotic ciprofloxacin showed superior efficacy with a zone of inhibition measuring 29.67 mm. The MIC values were determined as MIC_50_ at 0.328 ± 0.06 mg/mL and MIC_90_ at 0.384 ± 0.01 mg/mL. Furthermore, the minimal biofilm inhibition concentration, assessed using the crystal violet biofilm assay, was found to be MBIC_50_ at 0.377 ± 0.05 mg/mL and MBIC_90_ at 0.396 ± 0.03 mg/mL.

**Figure 2:**
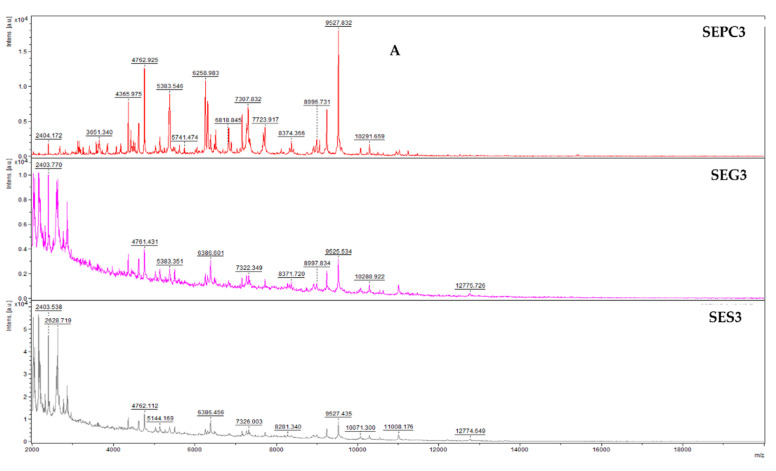

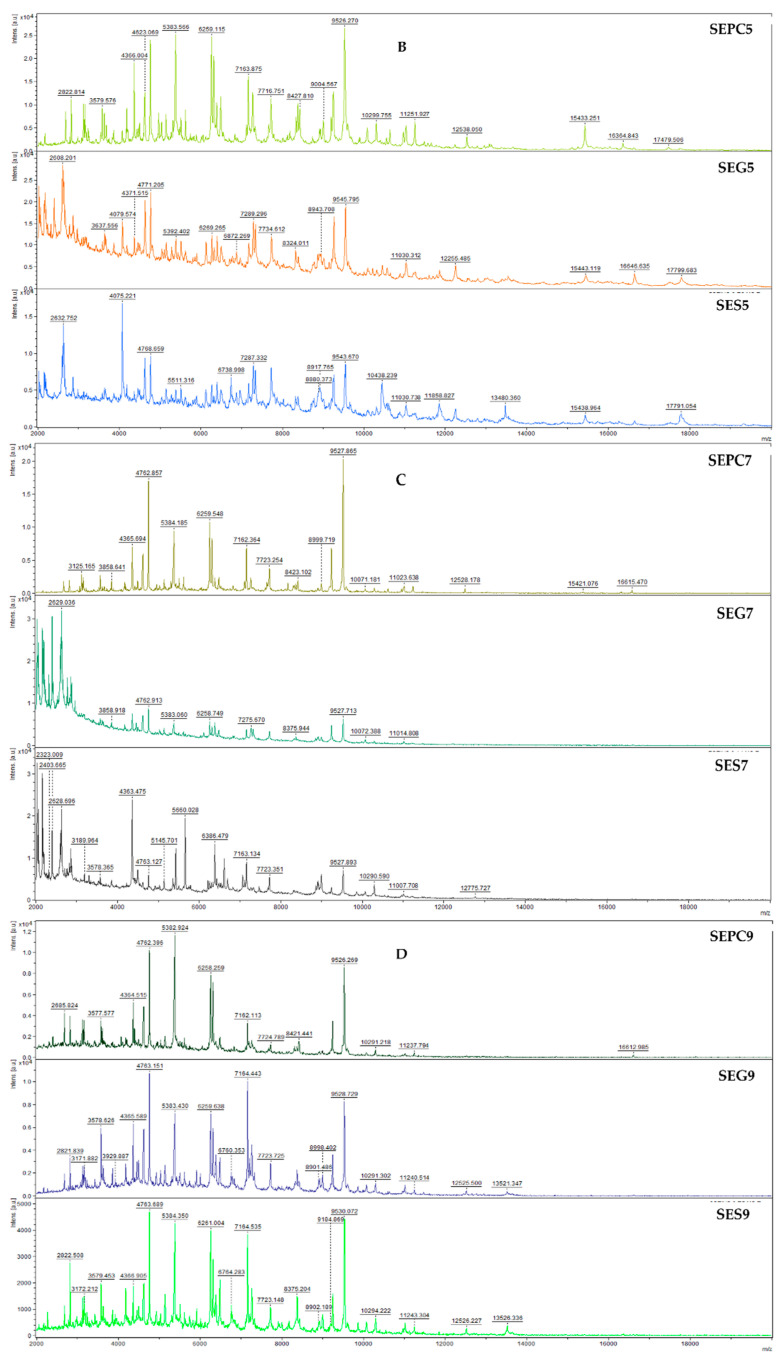

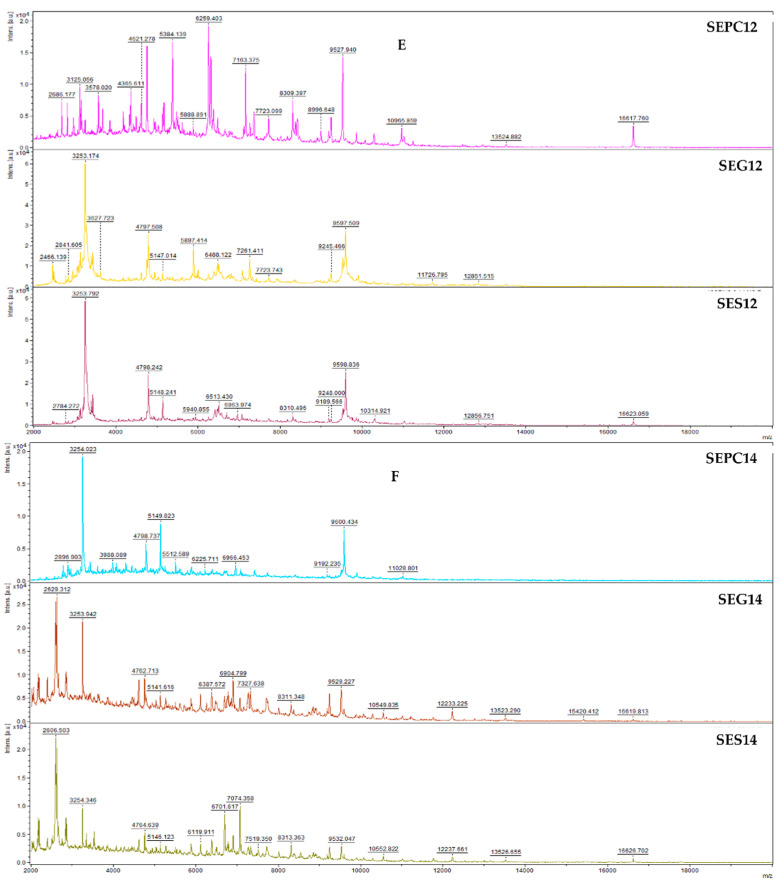
Representative MALDI-TOF mass spectra of *S. enterica*: (**A**) 3rd day; (**B**) 5th day; (**C**) 7th day; (**D**) 9th day; (**E**) 12th day; (**F**) 14th day. SE = *S. enterica*; G = glass; S = stainless-steel; and PC = planktonic cells.

Figure 2A–F depicts the MS spectra of different stages of *S. enterica* development treated with the ECEO on glass and stainless-steel surfaces. The spectra of planktonic cells, used as controls, are also presented. On the third day of treatment (SEPC 3, SEG 3, and SES 3), differences in protein spectra numbers were observed between the experimental and control groups, indicating some variation. However, similarities in spectrum evolution suggested that both groups were synthesizing similar proteins. By the fifth day (SEPC 5, SEG 5, and SES 5), distinct differences in mass spectrum evolution were noted between biofilms on both surfaces, suggesting the ECEO’s impact on biofilm stability. Significant variations were evident by the seventh day, particularly in the spectra of biofilms on plastic and stainless steel, indicating effective disruption by the ECEO. Nonetheless, some similarities in spectrum evolution persisted until the experiment’s conclusion. These findings demonstrate the ECEO’s ability to disrupt *S. enterica* biofilm homeostasis, with notable effects observed from day 3 to day 7 on both surfaces. This suggests that higher concentrations of the ECEO could potentially suppress biofilm development effectively over extended periods.

**Figure 3:**
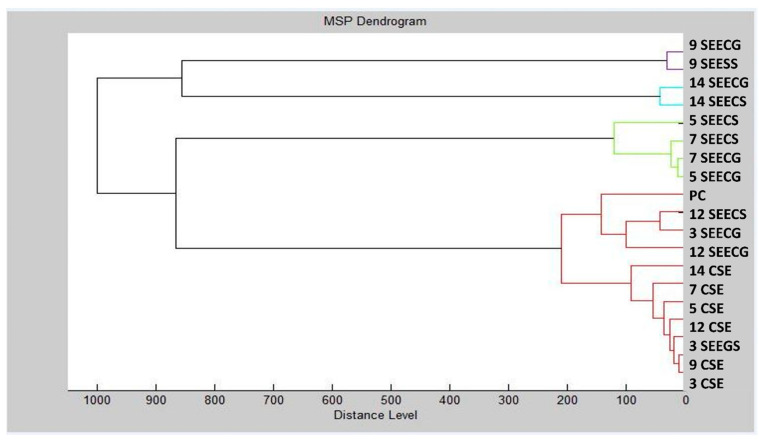
Dendrogram of *S. enterica* generated using MSPs of the planktonic cells and the control. SE = *S. enterica*; C = glass; S = stainless-steel; and PC = planktonic cells.

The dendrogram depicted in Figure 3 illustrates that on days 3 and 9, as well as during the early stages of biofilm formation on the experimental glass surface on days 5 and 7, the control groups exhibited the smallest MSP distances. Comparatively, the MSP distance was higher for the stainless-steel surface than for the glass surface, indicating that the ECEO had a more pronounced inhibitory effect on *S. enterica* biofilms on stainless steel. However, both glass and stainless-steel surfaces showed the greatest increase in MSP distance on days 9 and 14 of the experiment. Another aspect investigated was the minimal spectral peak (MSP) distances between planktonic cells and controls. Throughout the study, the MSP distances in the experimental group increased. Specifically, on the third day of the trial with the stainless-steel surface, the MSP distance in the experimental group was the shortest. By days 9 and 14, particularly on the plastic surface, the MSP distance for the experimental group had reached its maximum length. Similar trends were observed on days 5 and 7. Our study’s findings highlight the inhibitory and detrimental effects of the ECEO on *S. enterica* biofilm growth on both stainless steel and glass surfaces.

### 3.3. Sous Vide Red Deer Meet Microbiological Analyses

**Figure 4:**
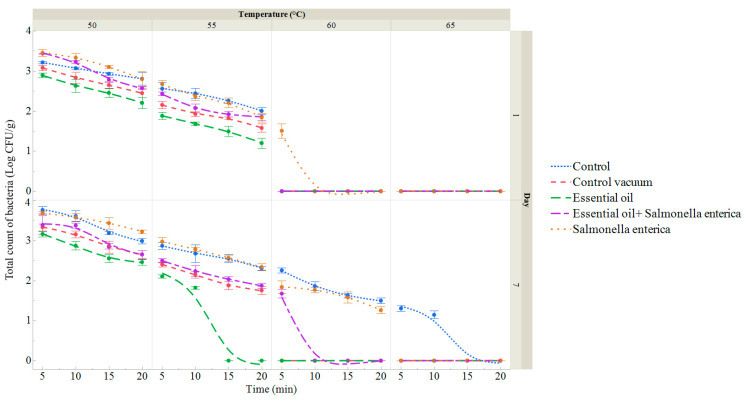
Total viable count (log CFU/g) of sous vide deer meat samples after 1 and 7 days of storage, treated in a water bath at temperatures between 50 and 65 °C for 5 to 20 min. Data are the mean (bars indicate ± SD) of 3 deer meat samples. Control: deer meat samples placed in polyethylene bags without vacuum. Control vacuum: deer meat samples vacuum-packed in polyethylene bags. Essential oil: deer meat samples treated with 1% ECEO and vacuum-packed. *Salmonella enterica*: deer meat samples inoculated with *S. enterica* and vacuum-packed. Essential oil + *Salmonella enterica:* deer meat samples treated with 1% ECEO and inoculated with *S. enterica* and vacuum-packed.

Figure 4 and Table S1 present the total viable count (TVC) of sous vide red deer meat samples subjected to different temperatures, times, the ECEO, and *S. enterica* treatments. Raw, uncooked, and unpackaged red deer meat served as control samples. Initial assessments on day 0 showed a TVC of 2.95 ± 0.06 log CFU/g with no presence of coliform bacteria.

On day 1, the TVC ranged from 2.00 log CFU/g (55 °C for 20 min) to 3.21 log CFU/g (50 °C for 5 min) in the sous vide red deer meat control group. Vacuum-packaged samples exhibited lower TVC compared to non-vacuum-packaged ones (Table S1). Specifically, vacuum-packaged samples ranged from 1.58 log CFU/g (55 °C for 20 min) to 3.08 log CFU/g (50 °C for 5 min), the ECEO-treated samples ranged from 1.20 log CFU/g (55 °C for 20 min) to 2.89 log CFU/g (50 °C for 5 min), and samples treated with both the ECEO and *S. enterica* ranged from 1.51 log CFU/g (60 °C for 5 min) to 3.44 log CFU/g (50 °C for 5 min). These results were recorded on the first day of storage.

By day 7, the TVC in the control group ranged from 1.14 log CFU/g (65 °C for 10 min) to 3.76 log CFU/g (50 °C for 5 min). Vacuum-packaged sous vide deer meat ranged from 1.75 log CFU/g (55 °C for 20 min) and the ECEO-treated samples ranged from 1.82 log CFU/g (55 °C for 10 min) to 3.16 log CFU/g (50 °C for 5 min) (Figure 4, Table S1). Samples treated solely with the ECEO on day 7 ranged from 1.82 log CFU/g (55 °C for 5 min) to 3.16 log CFU/g (50 °C for 5 min). For samples with S. enterica application, the TVC ranged from 1.26 log CFU/g (60 °C for 20 min) to 3.68 log CFU/g (50 °C for 5 min), and those treated with both the ECEO and *S. enterica* ranged from 1.67 log CFU/g.

**Figure 5:**
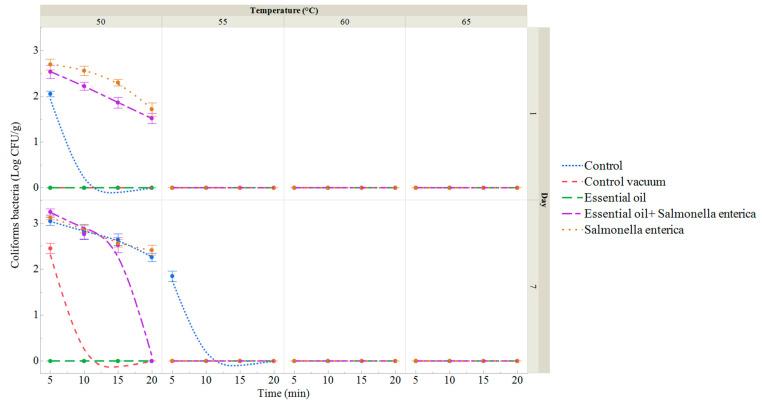
Total coliform bacteria (log CFU/g) of sous vide deer meat samples after 1 and 7 days of storage, treated in a water bath at temperatures between 50 and 65 °C for 5 to 20 min. Data are the mean (bars indicate ± SD) of 3 deer meat samples. Control: deer meat samples placed in polyethylene bags without vacuum. Control vacuum: deer meat samples vacuum-packed in polyethylene bags. Essential oil: deer meat samples treated with 1% ECEO and vacuum-packed. *Salmonella enterica*: deer meat samples inoculated with *S. enterica* and vacuum-packed. Essential oil + *Salmonella enterica:* deer meat samples treated with 1% ECEO and inoculated with *S. enterica* and vacuum-packed.

The number of coliform bacteria (CB) in samples of sous vide red deer meat is shown in Figure 5 and Table S2. On day 0, CB counts were zero. In the control group, packaged under aerobic conditions using polyethylene bags, CB were detected at 2.05 log CFU/g only in the first treatment of temperature and time. Coliform bacteria were first observed in sous vide deer meat samples on day seven. In the group where *S. enterica* was applied, CB ranged from 1.71 log CFU/g at a temperature treatment of 50 °C for 20 min to 2.69 log CFU/g at 50 °C for 5 min. In the group treated with the ECEO along with *S. enterica*, CB ranged from 1.52 log CFU/g at 50 °C for 20 min to 2.52 log CFU/g at 50 °C for 5 min. By day 7, CB counts in the control group ranged from 1.85 log CFU/g for the group treated for 5 min at 55 °C to 3.04 log CFU/g for the group treated for 5 min at 50 °C. In the vacuum-packed control group, CB counts ranged from 1.33 log CFU/g for the group treated at 55 °C for 5 min to 2.87 log CFU/g for the group treated at 50 °C for 5 min (Figure 5 and Table S2). In the group where the ECEO was applied, the number of CB was zero. On day 7 in the group with *S. enterica* application, CB ranged from 2.41 log CFU/g at 50 °C for 20 min to 3.12 log CFU/g at 50 °C for 5 min. CB counts ranged from 2.52 log CFU/g at 50 °C for 15 min to 3.25 log CFU/g in the group treated with both the ECEO and *S. enterica* application.

**Figure 6:**
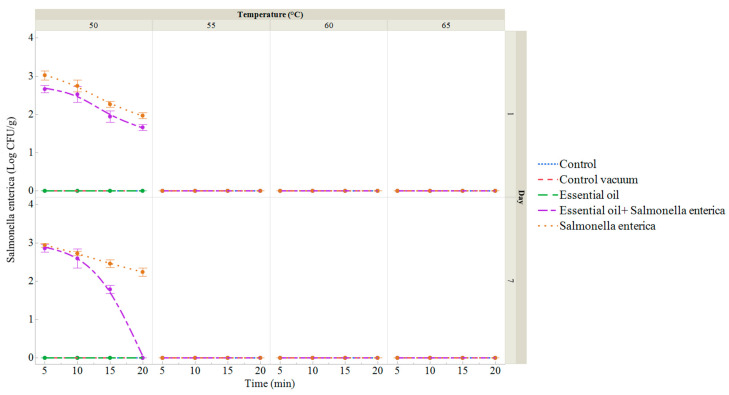
*Salmonella enterica* count (log CFU/g) of sous vide deer meat samples after 1 and 7 days of storage, treated in a water bath at temperatures between 50 and 65 °C for 5 to 20 min. Data are the mean (bars indicate ± SD) of 3 deer meat samples. Control: deer meat samples placed in polyethylene bags without vacuum. Control vacuum: deer meat samples vacuum-packed in polyethylene bags. Essential oil: deer meat samples treated with 1% ECEO and vacuum-packed. *Salmonella enterica*: deer meat samples inoculated with *S. enterica* and vacuum-packed. Essential oil + *Salmonella enterica:* deer meat samples treated with 1% ECEO and inoculated with *S. enterica* and vacuum-packed.

As depicted in Figure 6 and Table S3, *S. enterica* counts were only detected in the last two groups throughout the storage period. On day 1, counts in the group inoculated with *S. enterica* ranged from 1.97 log CFU/g (50 °C for 20 min) to 2.94 log CFU/g (50 °C for 5 min). In contrast, the group treated with the ECEO and inoculated with *S. enterica* showed counts ranging from 1.66 log CFU/g (50 °C for 20 min) to 2.66 log CFU/g (50 °C for 5 min) (Table S3). By day 7, counts in the *S. enterica* group ranged from 2.24 log CFU/g (50 °C for 20 min) to 2.94 log CFU/g (50 °C for 5 min), while in the group treated with the ECEO and inoculated with *S. enterica*, counts ranged from 1.78 log CFU/g (50 °C for 15 min) to 2.85 log CFU/g (50 °C for 5 min).

**Figure 7:**
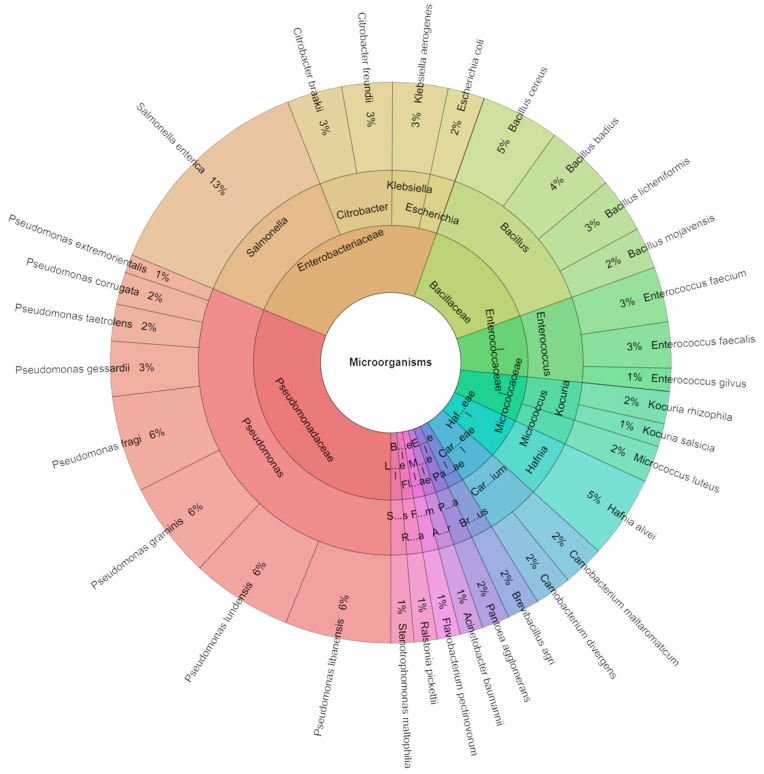
Krona chart: Isolated species, genera, and families from deer sous vide meat at 1 day.

Figure 7 displays species, genera, and families isolated from red deer sous vide meat samples on the first day of storage. A total of 377 isolates were identified using mass spectrometry with scores up to 2. These isolates belonged to 13 families, 17 genera, and 32 species. The most frequently identified species were from the families Pseudomonadaceae and Bacillaceae. The predominant species isolated from the sous vide deer meat samples on the first day included *S. enterica* (13%), which was intentionally inoculated into the meat, followed by *Pseudomonas fragi*, *Pseudomonas gessardii*, *Pseudomonas graminis*, *Pseudomonas libanensis*, and *Pseudomonas lundensis*, each with a frequency of 6%. *Hafnia alvei* and *Bacillus cereus* were also isolated with a frequency of 5% each.

**Figure 8:**
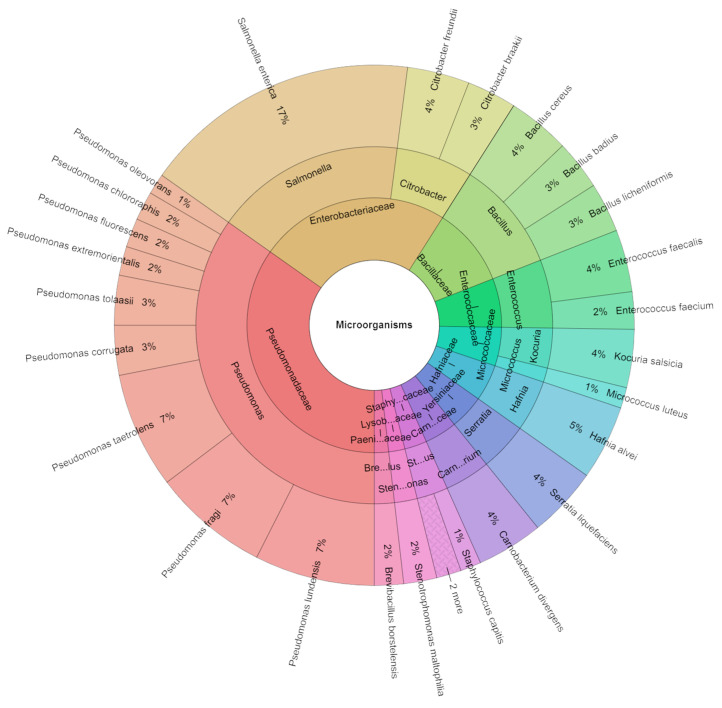
Krona chart: Isolated species, genera, and families from deer sous vide meat after 7 days.

Figure 8 shows species, genera, and families isolated from red deer sous vide meat samples after seven days of storage. A total of 388 isolates were identified using MALDI-TOF MS Biotyper with high scores. These isolates belonged to eleven families, thirteen genera, and twenty-seven species. The most frequently isolated family was Pseudomonadaceae, comprising nine species. The most commonly isolated species was *S. enterica* (17%) in the groups inoculated with this bacterium, followed by *Pseudomonas fragi*, *Pseudomonas lundensis*, and *Pseudomonas taetrolens*, each accounting for 7%.

## 4. Discussion

In our work, the ECEO consisted mainly of eugenol (82.7%). Comparing published data becomes challenging due to variations in EO composition, extraction methods, types of organisms tested, growth phases, inoculum volumes, culture media, pH levels, and incubation conditions, all of which can influence experimental outcomes [27,28,29]. Despite methodological differences, reported inhibition zones and minimum inhibitory concentrations (MICs) often show similarity across studies. Notably, much of the data focuses on *S. enterica*, a commonly used model organism [30]. Our study investigated the antimicrobial potential of clove essential oil against *S. enterica* using both the disc diffusion method and MIC determination. A different study indicates that the ECEO and its components are eugenol, β-caryophyllene, and acetyl eugenol, which exhibit significant antibacterial activity against a broad spectrum of microorganisms, encompassing Gram-positive and Gram-negative bacteria including *Streptococcus* species, *Staphylococcus aureus*, *Salmonella typhimurium*, *Escherichia coli*, *Klebsiella pneumoniae*, *Pseudomonas aeruginosa*, and *Agrobacterium* spp. Additionally, it effectively inhibits yeasts such as *Aspergillus* species, *Penicillium*, and *Candida albicans* [31]. Clove essential oil, extracted from the dried flower buds of the clove tree, is renowned for its antibacterial and antioxidant characteristics, primarily due to phenolic compounds such as eugenol. It has demonstrated effectiveness against foodborne pathogens, including *Salmonella*, *Escherichia coli*, *Staphylococcus*, and *Listeria* [32]. In our study, a 15.67 mm inhibition zone was observed for clove’s antimicrobial activity against *S. enterica*. In a different investigation, the zones of inhibition against *S. aureus*, *E. coli*, *L. monocytogenes*, and *S.* Typhimurium were measured as 2.83 cm, 2.81 cm, 2.47 cm, and 2.22 cm, respectively [33]. The existence and dimensions of inhibition zones serve as indicators of bacteria sensitivity to the essential oil. Typically, if the inhibition zone measures less than 0.7 cm, the sample is deemed inactive against the bacteria. Conversely, an inhibition zone diameter exceeding 1.2 cm indicates effective inhibitory efficacy [34]. Therefore, clove essential oil demonstrated effective inhibition against all tested bacteria in our study, consistent with its well-documented broad spectrum of inhibitory effects. The lipophilic properties of clove essential oil are likely responsible for disrupting bacterial cell membranes, thereby affecting their permeability [35]. Furthermore, our study revealed that clove essential oil exhibited inhibitory effects at concentrations as low as 0.328 mg/mL against *S. enterica*. According to Duarte et al. [36], essential oils with MICs up to 0.5 mg/mL are classified as having strong antimicrobial activity, while those with MICs between 0.6 and 1.5 mg/mL are considered moderate, and MICs above 1.6 mg/mL indicate weak activity. The MIC value of 0.328 mg/mL reported in our study aligns with the potent antibacterial activity noted by Silvestri et al. [37], who reported an MIC of 0.3 mg/mL for clove essential oil. For *Escherichia coli*, the MIC of clove essential oil in our study was 0.3047 mg/mL, lower than the range of 0.400–0.600 mg/mL reported by Silvestri et al. [37]. Similarly, the MIC for *S.* Typhimurium was lower in our study (0.0400 mg/mL) compared to values reported by Beraldo et al. [38]. MICs *for L. monocytogenes* were also lower than those reported by Beraldo et al. [38], where the MIC was 0.800 mg/mL. Discrepancies in MIC values across different studies may be attributed to variations in sample culture conditions, concentrations of components, and techniques used for essential oil extraction [39].

Microorganisms can develop heightened resistance to antimicrobial treatments when they form biofilms and intricate and spatially organized communities [40]. According to Zhao et al. [41], many pathogenic bacteria, including the *S. enterica* studied here, can cause illness through biofilm formation. Consequently, preventing the production and growth of biofilms poses a significant challenge in managing pathogenic bacteria and has become a pressing therapeutic concern [42,43]. Our study demonstrated strong antibiofilm activity using violet crystal assays. Another study using crystal violet staining highlighted the robust biofilm-forming capabilities of *Salmonella* Derby. Further investigation is required to explore the antibiofilm potential of EOs at sub-inhibitory concentrations from an economic perspective, and the specific mechanisms through which different EOs hinder *Salmonella* biofilm development remain unclear [44]. Clove essential oil, rich in eugenol, has been extensively researched for its ability to inhibit bacterial biofilms, including those formed by *Salmonella* Typhimurium, *Escherichia coli* O157, *Listeria monocytogenes*, and *Staphylococcus aureus* [42,45,46,47]. Although research has mainly focused on *S.* Typhimurium and *Salmonella* Enteritidis, there is limited exploration into the anti-biofilm effects of EOs on *Salmonella* Derby [48,49].

In our study, the ECEO, which has eugenol, β-caryophyllene, and acetyl eugenol as the main components, demonstrated effectiveness in disrupting the equilibrium of biofilms. However, from the beginning to the end of the experiment, there was a consistent resemblance in the progression of mass spectra between the experimental and control groups. Nonetheless, the data clearly indicate that the ECEO had a significant impact on disturbing the homeostasis of *S. enterica* biofilms. The sustained effectiveness of the ECEO on both surfaces suggests long-lasting benefits. In another study, the effect of the ECEO on the biofilms of *Bacillus subtilis* and *Stenotrophomonas maltophilia* was investigated using MALDI-TOF MS Biotyper to detect changes in molecular structures associated with growth suppression. To enhance clarity, the planktonic cell spectrum was substituted for the control spectrum, as the planktonic and biofilm spectra in the control group developed similarly. Each day during the experiment, spectra depicting planktonic growth in the control group and experimental spectra from various surfaces (wood and glass) were illustrated [25].

In our study, we assessed the microbiological quality of sous vide deer meat samples throughout a 7-day shelf-life period. Specifically, we assessed total counts and the presence of coliform bacteria and *Salmonella*. Our findings indicated that the microbial load decreased with higher temperatures and longer heat treatment times, with the lowest counts observed in groups treated with the ECEO. Clove essential oil demonstrated significant antibacterial activity against *S. enterica*, particularly under more intense heat treatments. Badei et al. [50] previously highlighted its effectiveness in inhibiting microbial growth in cookies, and it has been shown to combat both Gram-positive and Gram-negative bacteria, including reducing *E. coli* levels in ground beef and fermented sausages [51]. Recently, the ECEO has been employed as a natural food preservative and coloring agent due to its antibacterial properties and health benefits [52,53]. It has also been suggested as an alternative to acetic acid, sodium bicarbonate, and chlorine-based disinfectants for washing fresh-cut vegetables to mitigate microbial risks and extend shelf life [52,54]. Eugenol, constituting over 80% of the ECEO, primarily contributes to its antiseptic properties [55,56]. Studies by Latifah-Munirah et al. [55] and Rajkowska et al. [57] have demonstrated the effectiveness of eugenol and the ECEO against various pathogenic bacteria, including *S. enterica*, *S. aureus*, *E. coli*, *Proteus mirabilis*, *Pseudomonas aeruginosa*, and *Streptococcus mutans*. However, detailed investigations into the molecular antibacterial mechanisms of eugenol and the ECEO are limited, with current studies mainly focusing on their initial antibacterial activities [43,58]. Comprehensive studies, specifically on the effects of eugenol on foodborne microorganisms, are currently lacking. Nonetheless, due to its bactericidal, analgesic, antioxidant, and potential anti-cancer properties, the ECEO holds promise for diverse applications in the food and health sectors [59,60,61].

## 5. Conclusions

Our findings demonstrate the great effectiveness of 1.0% ECEO applied to deer meat combined with vacuum packaging against *Salmonella enterica*, coliform bacteria, and total viable count. Food shelf life is positively impacted, and food safety is improved when microorganisms in food are rendered inactive. As a natural antibacterial with a mild flavor, the ECEO can be used to keep vacuum-packaged deer meat fresher longer. Additional research is required to improve the inhibition of total viable numbers. This study concludes by highlighting the potential antibacterial and antibiofilm qualities of the ECEO, both *in vitro* and in relation to the preservation of meat. These characteristics point to its possible use in food preservation to prevent food spoiling and guarantee food safety, especially when paired with other modern packaging technologies and processing methods. *Salmonella enterica* contamination can be prevented by using the ECEO, which could prolong the shelf life of sous vide red deer meat while maintaining quality and safety standards.

## Figures and Tables

**Table 1:** Chemical composition (percentages mean values ± standard deviation) of the ECEO.

N°	Component ^1^	LRI ^2^	LRI ^3^	*Eugenia caryophyllus* EO ^4^
1	eugenol	1360	1363	82.7 ± 2.15
2	α-copaene	1365	1368	0.5 ± 0.02
3	isoeugenol	1441	1439	0.1 ± 0.01
4	β-caryophyllene	1460	1457	9.9 ± 0.18
5	humulene	1470	1466	1.3 ± 0.03
6	acetyl eugenol	1522	1525	3.4 ± 0.04
7	δ-cadinene	1530	1533	0.6 ± 0.03
8	trans-calamenene	1540	1536	0.5 ± 0.02
9	α-calacorene	1555	1560	0.1 ± 0.01
10	caryophyllene oxide	1608	1613	0.8 ± 0.02
11	humulene epoxide II	1618	1620	0.1 ± 0.00
	SUM			100.0
	Oxygenated Sesquiterpenes			0.9
	Hydrocarbon Sesquiterpenes			12.9
	Others			86.2

^1^ The components are reported according to their elution order on the apolar column; ^2^ Linear Retention indices measured on the apolar column; ^3^ Linear Retention indices from literature; ^4^ Percentage values of *Eugenia caryophyllus* components.

**Table 2:** Antimicrobial and antibiofilm activity of the *Eugenia caryophyllus* essential oil (ECEO) against *Salmonella enterica.* Data are presented as mean values ± standard deviation (SD) of three tests.

Inhibition Zone (mm)	ECEO	Ciprofloxacin
*Salmonella enterica*	15.67 ± 0.58	29.67 ± 0.56
Minimal inhibition concentration (mg/mL)	MIC_50_	MIC_90_
*Salmonella enterica*	0.328 ± 0.06	0.384 ± 0.01
Minimal biofilm inhibition concentration (mg/mL)	MBIC_50_	MBIC_90_
*Salmonella enterica*	0.377 ± 0.05	0.396 ± 0.03

## Data Availability

The original contributions presented in the study are included in the article/Supplementary Materials, further inquiries can be directed to the corresponding author.

## Supplementary Materials

The following supporting information can be downloaded at: https://www.mdpi.com/article/10.3390/foods13162512/s1, Table S1. Total viable count (log CFU/g) of sous vide red deer meat samples after storage 1, and 7 days treated in a water bath at temperatures between 50 and 65 °C for 5 to 20 min. Data are the mean (bars indicate ± SD) of 3 red deer meat samples; Table S2. Total coliform bacteria (log CFU/g) of sous vide red deer meat samples after storage 1, and 7 days treated in a water bath at temperatures between 50 and 65 °C for 5 to 20 min. Data are the mean (bars indicate ± SD) of 3 red deer meat samples; Table S3. *S. enterica* count (log CFU/g) of sous vide red deer meat samples after storage for 1 and 7 days treated in a water bath at temperatures between 50 and 65 °C for 5 to 20 min. Data are the mean (bars indicate ± SD) of 3 red deer meat samples.
