# Supplementary material for: Enhancing Deer Sous Vide Meat Shelf Life and Safety with Eugenia caryophyllus Essential Oil against Salmonella enterica
Source: Foods. 2024 Aug 12;13(16):2512. doi: 10.3390/foods13162512 (PMC11353597; doi:10.3390/foods13162512)
Supplement: Supplementary file 1 [file foods-13-02512-s001.zip › foods-3122991-supplementary.pdf]

Table S1. Total viable count (log CFU/g) of sous-vide deer meat samples after 1 and 7 days of storage, treated in a water bath at temperatures between 50 and 65 °C for 5 to 20 min. Control: deer meat samples placed in polyethylene bags without vacuum. Control vacuum: deer meat samples vacuum-packed in polyethylene bags. Essential oil: deer meat samples treated with 1% ECEO and vacuum-packed. *Salmonella enterica*: deer meat samples inoculated with *S. enterica* and vacuum-packed. Essential oil + *Salmonella enterica:* deer meat samples treated with 1% ECEO and inoculated with *S. enterica* and vacuum-packed.

Treatment	Temperature (°C)	Time (min)	Total count of bacteria (log CFU/g)	
			Days	
			1	7
Control	50	5	3.21±0.03^{b,A}	3.76±0.09^{a,A}
		10	3.07±0.04^{b,B}	3.61±0.14^{a,A}
		15	2.93±0.03^{b,C}	3.19±0.04^{a,B}
		20	2.81±0.16^{a,CD}	2.99±0.07^{a,C}
	55	5	2.56±0.17^{b,D}	2.87±0.09^{a,C}
		10	2.44±0.13^{a,DE}	2.67±0.22^{a,CD}
		15	2.26±0.07^{b,E}	2.55±0.09^{a,D}
		20	2.00±0.09^{b,F}	2.31±0.04^{a,E}
	60	5	n.d.^{b,G}	2.25±0.07^{a,E}
		10	n.d.^{b,G}	1.86±0.11^{a,F}
		15	n.d.^{b,G}	1.63±0.06^{a,G}
		20	n.d.^{b,G}	1.50±0.07^{a,G}
	65	5	n.d.^{b,G}	1.30±0.07^{a,H}
		10	n.d.^{b,G}	1.14±0.10^{a,I}
		15	n.d.^{a,G}	n.d.^{a,L}
		20	n.d.^{a,G}	n.d.^{a,L}
Control vacuum	50	5	3.08±0.07^{b,A}	3.33±0.09^{a,A}
		10	2.83±0.14^{b,B}	3.15±0.08^{a,B}
		15	2.65±0.10^{a,BC}	2.84±0.15^{a,C}
		20	2.45±0.11^{a,C}	2.65±0.11^{a,C}
	55	5	2.15±0.09^{b,D}	2.40±0.06^{a,D}
		10	1.93±0.06^{b,E}	2.15±0.09^{a,E}
		15	1.82±0.04^{a,F}	1.88±0.10^{a,F}
		20	1.58±0.11^{a,G}	1.75±0.11^{a,F}
	60	5	n.d.^{a,H}	n.d.^{a,G}
		10	n.d.^{a,H}	n.d.^{a,G}
		15	n.d.^{a,H}	n.d.^{a,G}
		20	n.d.^{a,H}	n.d.^{a,G}
	65	5	n.d.^{a,H}	n.d.^{a,G}
		10	n.d.^{a,H}	n.d.^{a,G}
		15	n.d.^{a,H}	n.d.^{a,G}
		20	n.d.^{a,H}	n.d.^{a,G}
Essential oil	50	5	2.89±0.06^{b,A}	3.16±0.07^{a,A}
		10	2.63±0.16^{a,B}	2.87±0.10^{a,B}
		15	2.45±0.12^{a,BC}	2.55±0.10^{a,C}
		20	2.20±0.14^{b,C}	2.46±0.07^{a,C}
	55	5	1.88±0.10^{b,D}	2.10±0.04^{a,D}
		10	1.68±0.05^{b,E}	1.82±0.04^{a,E}
		15	1.49±0.13^{a,E}	n.d.^{b,F}
		20	1.20±0.12^{a,F}	n.d.^{b,F}

	60	5	n.d.^{a,G}	n.d.^{a,F}
		10	n.d.^{a,G}	n.d.^{a,F}
		15	n.d.^{a,G}	n.d.^{a,F}
		20	n.d.^{a,G}	n.d.^{a,F}
	65	5	n.d.^{a,G}	n.d.^{a,F}
		10	n.d.^{a,G}	n.d.^{a,F}
		15	n.d.^{a,G}	n.d.^{a,F}
		20	n.d.^{a,G}	n.d.^{a,F}
Salmonella enterica	50	5	3.45±0.09^{b,A}	3.68±0.04^{a,A}
		10	3.33±0.11^{b,A}	3.58±0.10^{a,AB}
		15	3.10±0.04^{b,B}	3.43±0.15^{a,BC}
		20	2.80±0.19^{b,C}	3.22±0.05^{a,C}
	55	5	2.67±0.10^{b,C}	2.97±0.11^{a,D}
		10	2.36±0.10^{b,D}	2.78±0.05^{a,E}
		15	2.21±0.12^{b,D}	2.57±0.09^{a,F}
		20	1.84±0.11^{b,E}	2.34±0.08^{a,G}
	60	5	1.51±0.18^{a,F}	1.84±0.16^{a,H}
		10	n.d.^{b,G}	1.77±0.07^{a,H}
		15	n.d.^{b,G}	1.58±0.14^{a,H}
		20	n.d.^{b,G}	1.26±0.09^{a,I}
	65	5	n.d.^{a,G}	n.d.^{a,L}
		10	n.d.^{a,G}	n.d.^{a,L}
		15	n.d.^{a,G}	n.d.^{a,L}
		20	n.d.^{a,G}	n.d.^{a,L}
Essential oil+ *Salmonella enterica*	50	5	3.44±0.02^{a,A}	3.39±0.23^{a,A}
		10	3.23±0.03^{b,B}	3.37±0.07^{a,A}
		15	2.78±0.07^{a,C}	2.86±0.07^{a,B}
		20	2.57±0.04^{a,D}	2.65±0.11^{a,C}
	55	5	2.43±0.04^{a,E}	2.50±0.06^{a,C}
		10	2.07±0.11^{a,F}	2.23±0.14^{a,D}
		15	1.92±0.07^{a,FG}	2.03±0.06^{a,E}
		20	1.86±0.09^{a,G}	1.87±0.06^{a,F}
	60	5	n.d.^{b,H}	1.67±0.11^{a,G}
		10	n.d.^{a,H}	n.d.^{a,H}
		15	n.d.^{a,H}	n.d.^{a,H}
		20	n.d.^{a,H}	n.d.^{a,H}
	65	5	n.d.^{a,H}	n.d.^{a,H}
		10	n.d.^{a,H}	n.d.^{a,H}
		15	n.d.^{a,H}	n.d.^{a,H}
		20	n.d.^{a,H}	n.d.^{a,H}

Values are presented as mean ± standard deviation (SD) of of 3 deer meat samples. $^{a\text{-}b}$ Differents superscripts lowercase letters indicate statistical different value within row (Duncan's MRT, $p \le 0.05$). $^{A\text{-}L}$ Differents superscripts uppercase letters indicate statistical different value within column for each treatment (Duncan's MRT, $p \le 0.05$). n.d.= not detected (value = 0.00).

Table S2. Total coliforms bacteria (log CFU/g) of sous-vide deer meat samples after 1 and 7 days of storage, treated in a water bath at temperatures between 50 and 65 °C for 5 to 20 min. Control: deer meat samples placed in polyethylene bags without vacuum. Control vacuum: deer meat samples vacuum-packed in polyethylene bags. Essential oil: deer meat samples treated with 1% ECEO and vacuum-packed. *Salmonella enterica*: deer meat samples inoculated with *S. enterica* and vacuum-packed. Essential oil + *Salmonella enterica:* deer meat samples treated with 1% ECEO and inoculated with *S. enterica* and vacuum-packed.

Treatment	Temperature (°C)	Time (min)	Coliforms bacteria (log CFU/g)	
			Days	
			1	7
Control	50	5	2.05±0.07^{b,A}	3.04±0.09^{a,A}
		10	n.d.^{b,B}	2.81±0.16^{a,AB}
		15	n.d.^{b,B}	2.64±0.13^{a,B}
		20	n.d.^{b,B}	2.26±0.09^{a,C}
	55	5	n.d.^{b,B}	1.85±0.11^{a,D}
		10	n.d.^{a,B}	n.d.^{a,E}
		15	n.d.^{a,B}	n.d.^{a,E}
		20	n.d.^{a,B}	n.d.^{a,E}
	60	5	n.d.^{a,B}	n.d.^{a,E}
		10	n.d.^{a,B}	n.d.^{a,E}
		15	n.d.^{a,B}	n.d.^{a,E}
		20	n.d.^{a,B}	n.d.^{a,E}
	65	5	n.d.^{a,B}	n.d.^{a,E}
		10	n.d.^{a,B}	n.d.^{a,E}
		15	n.d.^{a,B}	n.d.^{a,E}
		20	n.d.^{a,B}	n.d.^{a,E}
Control vacuum	50	5	n.d.^{b,A}	2.45±0.11^{a,A}
		10	n.d.^{a,A}	n.d.^{a,B}
		15	n.d.^{a,A}	n.d.^{a,B}
		20	n.d.^{a,A}	n.d.^{a,B}
	55	5	n.d.^{a,A}	n.d.^{a,B}
		10	n.d.^{a,A}	n.d.^{a,B}
		15	n.d.^{a,A}	n.d.^{a,B}
		20	n.d.^{a,A}	n.d.^{a,B}
	60	5	n.d.^{a,A}	n.d.^{a,B}
		10	n.d.^{a,A}	n.d.^{a,B}
		15	n.d.^{a,A}	n.d.^{a,B}
		20	n.d.^{a,A}	n.d.^{a,B}
	65	5	n.d.^{a,A}	n.d.^{a,B}
		10	n.d.^{a,A}	n.d.^{a,B}
		15	n.d.^{a,A}	n.d.^{a,B}
		20	n.d.^{a,A}	n.d.^{a,B}
Essential oil	50	5	n.d.^{a,A}	n.d.^{a,A}
		10	n.d.^{a,A}	n.d.^{a,A}
		15	n.d.^{a,A}	n.d.^{a,A}
		20	n.d.^{a,A}	n.d.^{a,A}
	55	5	n.d.^{a,A}	n.d.^{a,A}
		10	n.d.^{a,A}	n.d.^{a,A}
		15	n.d.^{a,A}	n.d.^{a,A}
		20	n.d.^{a,A}	n.d.^{a,A}

	60	5	n.d.^{a,A}	n.d.^{a,A}
		10	n.d.^{a,A}	n.d.^{a,A}
		15	n.d.^{a,A}	n.d.^{a,A}
		20	n.d.^{a,A}	n.d.^{a,A}
	65	5	n.d.^{a,A}	n.d.^{a,A}
		10	n.d.^{a,A}	n.d.^{a,A}
		15	n.d.^{a,A}	n.d.^{a,A}
		20	n.d.^{a,A}	n.d.^{a,A}
Salmonella enterica	50	5	2.69±0.12^{b,A}	3.12±0.04^{a,A}
		10	2.56±0.10^{b,A}	2.87±0.09^{a,B}
		15	2.30±0.08^{b,B}	2.56±0.08^{a,CD}
		20	1.71±0.14^{b,C}	2.41±0.11^{a,D}
	55	5	n.d.^{a,D}	n.d.^{a,E}
		10	n.d.^{a,D}	n.d.^{a,E}
		15	n.d.^{a,D}	n.d.^{a,E}
		20	n.d.^{a,D}	n.d.^{a,E}
	60	5	n.d.^{a,D}	n.d.^{a,E}
		10	n.d.^{a,D}	n.d.^{a,E}
		15	n.d.^{a,D}	n.d.^{a,E}
		20	n.d.^{a,D}	n.d.^{a,E}
	65	5	n.d.^{a,D}	n.d.^{a,E}
		10	n.d.^{a,D}	n.d.^{a,E}
		15	n.d.^{a,D}	n.d.^{a,E}
		20	n.d.^{a,D}	n.d.^{a,E}
Essential oil+ *Salmonella enterica*	50	5	2.53±0.14^{b,A}	3.25±0.07^{a,A}
		10	2.22±0.09^{b,B}	2.76±0.10^{a,B}
		15	1.86±0.12^{b,C}	2.52±0.17^{a,B}
		20	1.52±0.12^{a,D}	n.d.^{b,C}
	55	5	n.d.^{a,E}	n.d.^{a,C}
		10	n.d.^{a,E}	n.d.^{a,C}
		15	n.d.^{a,E}	n.d.^{a,C}
		20	n.d.^{a,E}	n.d.^{a,C}
	60	5	n.d.^{a,E}	n.d.^{a,C}
		10	n.d.^{a,E}	n.d.^{a,C}
		15	n.d.^{a,E}	n.d.^{a,C}
		20	n.d.^{a,E}	n.d.^{a,C}
	65	5	n.d.^{a,E}	n.d.^{a,C}
		10	n.d.^{a,E}	n.d.^{a,C}
		15	n.d.^{a,E}	n.d.^{a,C}
		20	n.d.^{a,E}	n.d.^{a,C}

Values are presented as mean ± standard deviation (SD) of of 3 deer meat samples. $^{a-b}$ Differents superscripts lowercase letters indicate statistical different value within row (Duncan's MRT, $p \leq 0.05$). $^{A-E}$ Differents superscripts uppercase letters indicate statistical different value within column for each treatment (Duncan's MRT, $p \leq 0.05$). n.d.= not detected (value = 0.00).

Table S3. *Salmonella enterica* count (log CFU/g) of sous-vide deer meat samples after 1 and 7 days of storage, treated in a water bath at temperatures between 50 and 65 °C for 5 to 20 min. Control: deer meat samples placed in polyethylene bags without vacuum. Control vacuum: deer meat samples vacuum-packed in polyethylene bags. Essential oil: deer meat samples treated with 1% ECEO and vacuum-packed. *Salmonella enterica*: deer meat samples inoculated with *S. enterica* and vacuum-packed. Essential oil + *Salmonella enterica:* deer meat samples treated with 1% ECEO and inoculated with *S. enterica* and vacuum-packed.

**Treatment**	**Temperature (°C)**	**Time (min)**	***Salmonella enterica* (log CFU/g)**	
			**Days**	
			**1**	**7**
Control	50	5	n.d.^{a,A}	n.d.^{a,A}
		10	n.d.^{a,A}	n.d.^{a,A}
		15	n.d.^{a,A}	n.d.^{a,A}
		20	n.d.^{a,A}	n.d.^{a,A}
	55	5	n.d.^{a,A}	n.d.^{a,A}
		10	n.d.^{a,A}	n.d.^{a,A}
		15	n.d.^{a,A}	n.d.^{a,A}
		20	n.d.^{a,A}	n.d.^{a,A}
	60	5	n.d.^{a,A}	n.d.^{a,A}
		10	n.d.^{a,A}	n.d.^{a,A}
		15	n.d.^{a,A}	n.d.^{a,A}
		20	n.d.^{a,A}	n.d.^{a,A}
	65	5	n.d.^{a,A}	n.d.^{a,A}
		10	n.d.^{a,A}	n.d.^{a,A}
		15	n.d.^{a,A}	n.d.^{a,A}
		20	n.d.^{a,A}	n.d.^{a,A}
Control vacuum	50	5	n.d.^{a,A}	n.d.^{a,A}
		10	n.d.^{a,A}	n.d.^{a,A}
		15	n.d.^{a,A}	n.d.^{a,A}
		20	n.d.^{a,A}	n.d.^{a,A}
	55	5	n.d.^{a,A}	n.d.^{a,A}
		10	n.d.^{a,A}	n.d.^{a,A}
		15	n.d.^{a,A}	n.d.^{a,A}
		20	n.d.^{a,A}	n.d.^{a,A}
	60	5	n.d.^{a,A}	n.d.^{a,A}
		10	n.d.^{a,A}	n.d.^{a,A}
		15	n.d.^{a,A}	n.d.^{a,A}
		20	n.d.^{a,A}	n.d.^{a,A}
	65	5	n.d.^{a,A}	n.d.^{a,A}
		10	n.d.^{a,A}	n.d.^{a,A}
		15	n.d.^{a,A}	n.d.^{a,A}
		20	n.d.^{a,A}	n.d.^{a,A}
Essential oil	50	5	n.d.^{a,A}	n.d.^{a,A}
		10	n.d.^{a,A}	n.d.^{a,A}
		15	n.d.^{a,A}	n.d.^{a,A}
		20	n.d.^{a,A}	n.d.^{a,A}
	55	5	n.d.^{a,A}	n.d.^{a,A}
		10	n.d.^{a,A}	n.d.^{a,A}
		15	n.d.^{a,A}	n.d.^{a,A}
		20	n.d.^{a,A}	n.d.^{a,A}

	60	5	n.d.^{a,A}	n.d.^{a,A}
		10	n.d.^{a,A}	n.d.^{a,A}
		15	n.d.^{a,A}	n.d.^{a,A}
		20	n.d.^{a,A}	n.d.^{a,A}
	65	5	n.d.^{a,A}	n.d.^{a,A}
		10	n.d.^{a,A}	n.d.^{a,A}
		15	n.d.^{a,A}	n.d.^{a,A}
		20	n.d.^{a,A}	n.d.^{a,A}
Salmonella enterica	50	5	3.02±0.12^{a,A}	2.94±0.05^{a,A}
		10	2.74±0.16^{a,A}	2.72±0.06^{a,B}
		15	2.26±0.08^{a,B}	2.45±0.10^{a,C}
		20	1.97±0.08^{b,C}	2.24±0.10^{a,D}
	55	5	n.d.^{a,D}	n.d.^{a,E}
		10	n.d.^{a,D}	n.d.^{a,E}
		15	n.d.^{a,D}	n.d.^{a,E}
		20	n.d.^{a,D}	n.d.^{a,E}
	60	5	n.d.^{a,D}	n.d.^{a,E}
		10	n.d.^{a,D}	n.d.^{a,E}
		15	n.d.^{a,D}	n.d.^{a,E}
		20	n.d.^{a,D}	n.d.^{a,E}
	65	5	n.d.^{a,D}	n.d.^{a,E}
		10	n.d.^{a,D}	n.d.^{a,E}
		15	n.d.^{a,D}	n.d.^{a,E}
		20	n.d.^{a,D}	n.d.^{a,E}
Essential oil+ *Salmonella enterica*	50	5	2.66±0.10^{b,A}	2.85±0.10^{a,A}
		10	2.52±0.21^{a,A}	2.59±0.25^{a,A}
		15	1.94±0.15^{a,B}	1.78±0.11^{a,B}
		20	1.66±0.08^{a,C}	n.d.^{a,C}
	55	5	n.d.^{a,D}	n.d.^{a,C}
		10	n.d.^{a,D}	n.d.^{a,C}
		15	n.d.^{a,D}	n.d.^{a,C}
		20	n.d.^{a,D}	n.d.^{a,C}
	60	5	n.d.^{a,D}	n.d.^{a,C}
		10	n.d.^{a,D}	n.d.^{a,C}
		15	n.d.^{a,D}	n.d.^{a,C}
		20	n.d.^{a,D}	n.d.^{a,C}
	65	5	n.d.^{a,D}	n.d.^{a,C}
		10	n.d.^{a,D}	n.d.^{a,C}
		15	n.d.^{a,D}	n.d.^{a,C}
		20	n.d.^{a,D}	n.d.^{a,C}

Values are presented as mean ± standard deviation (SD) of of 3 deer meat samples. $^{a\text{-}b}$ Differents superscripts lowercase letters indicate statistical different value within row (Duncan's MRT, $p \leq 0.05$). $^{A\text{-}E}$ Differents superscripts uppercase letters indicate statistical different value within column for each treatment (Duncan's MRT, $p \leq 0.05$). n.d.= not detected (value = 0.00).
